# Females have an increased risk of short‐term mortality after cardiac surgery compared to males: Insights from a national database

**DOI:** 10.1111/jocs.16928

**Published:** 2022-09-18

**Authors:** Lauren Kari Dixon, Arnaldo Dimagli, Ettorino Di Tommaso, Shubhra Sinha, Daniel P. Fudulu, Manraj Sandhu, Umberto Benedetto, Gianni D. Angelini

**Affiliations:** ^1^ Bristol Heart Institute University of Bristol Bristol UK

**Keywords:** AVR, CABG, cardiac surgery, disparities, gender, mortality, MVR, sex

## Abstract

**Objectives:**

Female sex is considered a risk factor for mortality and morbidity following cardiac surgery. This study is the first to review the UK adult cardiac surgery national database to compare outcomes following surgical coronary revascularisation and valvular procedures between females and males.

**Methods:**

Using data from National Adult Cardiac Surgery Audit, we identified all elective and urgent, isolated coronary artery by‐pass grafting (CABG), aortic valve replacement (AVR) and mitral valve replacement/repair (MVR) procedures from 2010 to 2018. We compared baseline data, operative data and outcomes of mortality, stroke, renal failure, deep sternal wound infection, return to theater for bleeding, and length of hospital stay. Multivariable mixed‐effect logistical/linear regression models were used to assess relationships between sex and outcomes, adjusting for baseline characteristics.

**Results:**

Females, compared to males, had greater odds of experiencing 30‐day mortality (CABG odd ratio [OR] 1.76, confidence interval [CI] 1.47−2.09, *p* < .001; AVR OR 1.59, CI 1.27−1.99, *p* < .001; MVR OR 1.37, CI 1.09−1.71, *p* = .006). After CABG, females also had higher rates of postoperative dialysis (OR 1.31, CI 1.12−1.52, *p* < .001), deep sternal wound infections (OR 1.43, CI 1.11−1.83, *p* = .005) and longer length of hospital stay (*β* 1.2, CI 1.0−1.4, *p* < .001) compared to males. Female sex was protective against returning to theater for postoperative bleeding following CABG (OR 0.76, CI 0.65−0.87, *p* < .001) and AVR (OR 0.72, CI 0.61−0.84, *p* < .001).

**Conclusion:**

Females in the United Kingdom have an increased risk of short‐term mortality after cardiac surgery compared to males. This highlights the need to focus on the understanding of the causes behind these disparities and implementation of strategies to improve outcomes in females.

## Key Points

- This study is the first to review current practice of UK national data to compare sex‐related differences in outcomes following surgical coronary revascularisation and valvular cardiac procedures.
- A large data set of 210,155 patients (25.7% female).
- Females have a higher risk of short‐term mortality following CABG, AVR and MVR in the UK.

**Abbreviations**
AF: atrial fibrillation
AVR: aortic valve replacement
BIMA: bilateral internal mammary arteries
BMI: body mass index
CABG: coronary artery bypass graft
CI: confidence interval
COPD: chronic obstructive pulmonary disease
CPB: cardio‐pulmonary bypass
HR: hazard ratio
IQR: interquartile range
LIMA: left internal mammary artery
MI: myocardial infarction
MVR: mitral valve repair or replacement
NACSA: National Adult Cardiac Surgery Audit
NICOR: National Institute of Cardiovascular Outcomes Research
ONS: Office for National Statistics
OR: odds ratio
RIMA: right internal mammary artery
SD: standard deviation
SSI: surgical site infection

## 1 INTRODUCTION

Female sex is reported as a risk factor for mortality and morbidity during and after cardiac surgery.^1^ Whilst advancements in technology and perioperative care have improved cardiac surgery mortality overall, risk prediction models such as Society of Thoracic Surgeons (STS) and Euroscore II, still confer additional operative risk to female patients.^2^

Many explanations for this increased peri‐operative risk have been postulated. First, women tend to present later in their disease process and have poorer preoperative baseline risk profiles than males.^3^ Second, female anatomy is reported to be operatively more challenging with smaller, tortuous coronary arteries, and smaller diameter cardiac valves.^4^, ^5^

A meta‐analysis previously published by our group showed females were at a higher risk of mortality and postoperative stroke than males following cardiac surgery.^6^ Findings from many observational national datasets support this conclusion.^7^, ^8^, ^9^, ^10^ A national‐level study from Sweden attributed the higher mortality seen in females to increased burden of risk factors rather than female sex as an independent risk factor.^7^ A similar study from Australia supported this conclusion.^10^ A study of Italian hospitals showed a higher mortality rate in females following coronary artery bypass surgery which was explained by technically more challenging coronary surgery in women.^9^ A retrospective analysis of a nationwide database from United States showed that whilst woman continue to have higher rates of mortality after Coronary artery by pass graft (CABG), the gender gap is slowly closing.^8^ However, analyses of other national databases have not reported sex‐related differences in mortality following cardiac surgery.^11^, ^12^ An Australian study of combined aortic valve replacement (AVR) and CABG showed equal short‐term and long‐term outcomes between sexes.^11^ An analysis of AVR performed in Canada did not find females to have a high risk of mortality and in fact showed female sex to be protective against postoperative stroke.^12^ To date, there has not been a sex‐specific analysis of contemporary practice from the United Kingdom.

The aim of this study, therefore, was to determine the differences in outcomes between females and males after coronary revascularisation and valvular cardiac surgery within the United Kingdom's current practice. The primary outcome was short‐term (30‐day) mortality. Secondary outcomes included short‐term complications such as stroke, sternal wound infection, reoperation for bleeding and length of hospital stay.

## 2 MATERIALS AND METHODS

A complete extract of prospectively collected data from the National Adult Cardiac Surgery Audit (NACSA) was obtained from the National Institute of Cardiovascular Outcomes Research (NICOR) central cardiac database and retrospectively analyzed. The definitions of the database variables used for this study are available at https://www.nicor.org.uk/national-cardiac-audit-programme/adult-cardiac-surgery-surgery-audit/. The NICOR registry prospectively collects demographic, pre and postoperative clinical information, including mortality, for all major adult cardiac surgery procedures performed in the United Kingdom. The flow of the data from surgeon‐input to analysis has been described elsewhere.^13^ Briefly, data entered locally by surgeons are validated at the unit‐level by database managers before upload NICOR. At this stage, further validation is performed according to logical rules and missing data reports are generated for primary variables (e.g., EuroSCORE risk factors, patient identifiers and outcome data). The data are cleaned by the academic healthcare informatics department. The complete data cleaning process has been previously described.^13^ Duplicate records are removed, transcriptional discrepancies re‐coded and clinical and temporal conflicts resolved. Missing data are resolved during the validation stages of the data transfer from individual centers. Missing and conflicting data for in‐hospital mortality status are backfilled and validated via record linkage to the Office for National Statistics census database. The overall percentage of missing data for baseline information is very low (1.7%). Missing categorical or dichotomous variable data were imputed with the mode while missing continuous variables data imputed with the median.

For this study, we used the NACSA data set to include all adult patients who underwent cardiac surgery between 2010 and 2018. We included elective and urgent isolated coronary revascularization and valvular procedures (AVR, mitral valve replacement/repair [MVR]). We excluded minor procedures, aortic arch surgery, heart transplantation and emergency, and salvage surgery.

### 2.1 Statistical analysis

A Shapiro−Wilks test was used to assess normality of distribution of continuous data. Data of normal distribution was averaged as a mean with standard deviation and analyzed using student *t*‐test. Nonnormally distributed data was averaged as a median with interquartile range and analyzed using a rank sum test. Categorical data is presented as frequencies and compared using a *χ* ^2^ test. For binary outcomes, a logistical regression model was used. For continuous outcomes, a linear regression model was used. Multivariable analysis was used to assess relationships between sex and our outcomes, adjusting for baseline characteristics including age, body mass index (BMI), smoking status, diabetes, chronic obstructive pulmonary disease (COPD), renal failure, cerebrovascular disease, peripheral vascular disease, atrial fibrillation, and hypertension. *p* < .05 was considered significant in all the analysis. Statistical analysis was performed using R version 4.0.0 using the packages sjplot, lme4, lmertest, gtsummary, and ggplot2.

## 3 RESULTS

### 3.1 Patient characteristics

**Figure 1:**
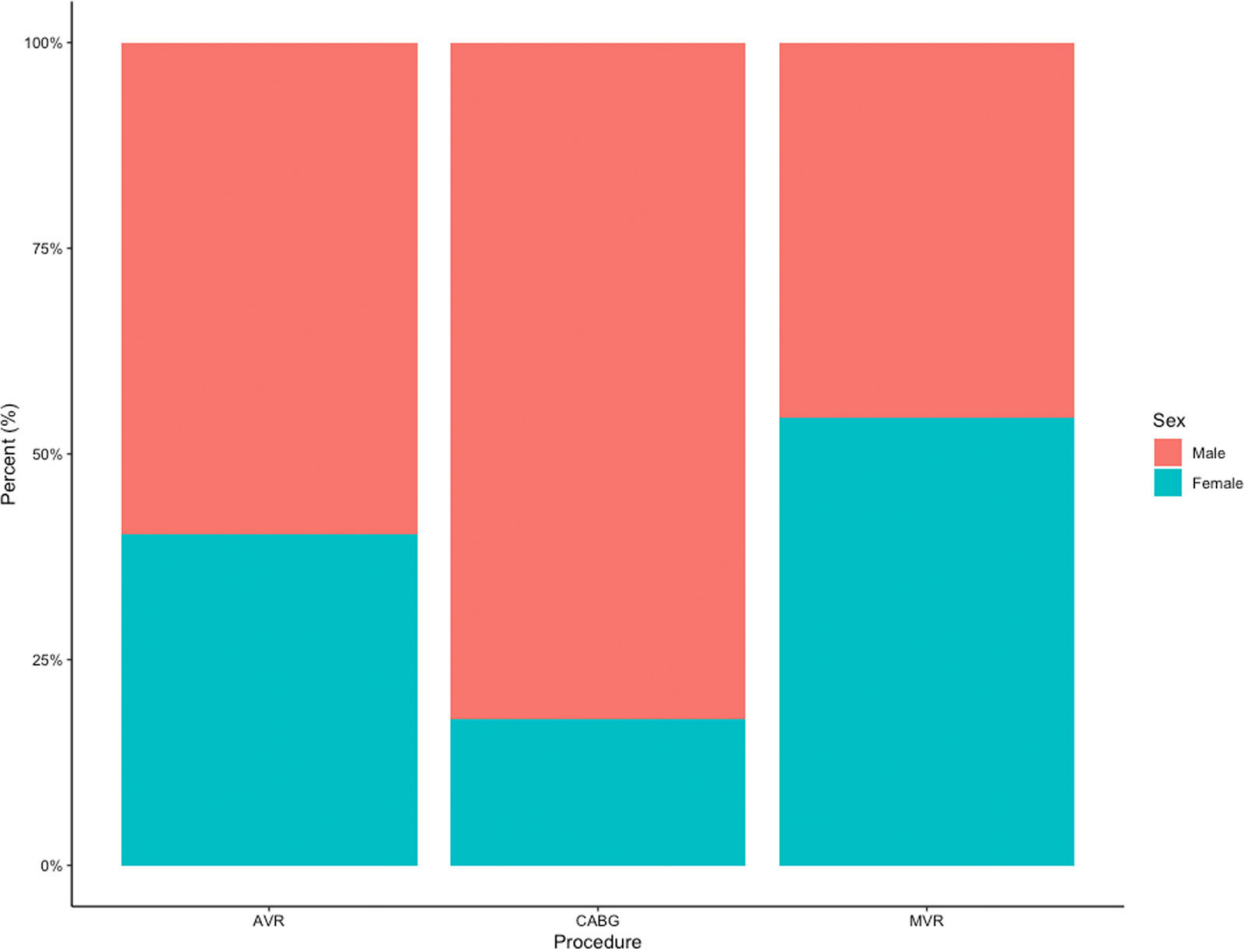
Percentage of procedure AVR, CABG and MVR by sex. AVR, aortic valve replacement; CABG, coronary artery bypass graft; MVR, mitral valve replacement/repair.

CABG and AVR were more commonly performed in males than females whereas more females underwent MVR than males (Figure 1 shows the proportion of each intervention per sex).

### 3.2 CABG

During the study period, 121,319 males (82.3%) and 26,157 females (17.7%) underwent an elective or urgent isolated CABG (see Table 1). Females tended to be older with more comorbidities such as diabetes, COPD and hypertension. Females were more likely than males to have an urgent rather than elective CABG and females scored more highly in New York Heart Association and Canadian Cardiovascular Society  scoring systems. Females had shorter cross clamp times and cardio‐pulmonary bypass (CPB) times (see Table 2). Male were more likely to receive internal mammary artery grafts including left internal mammary artery (LIMA), right internal mammary artery (RIMA) or bilateral internal mammary arteries (BIMA) than females. However, a higher proportion of females received total arterial grafts than males.

**Table 1:** Baseline characteristics of patients undergoing CABG, females compared to males

Characteristic	Male, *N* = 121,319^a^	Female, *N* = 26,157^a^	*p* Value^b^
Age (years)	67 (60−74)	67 (60−74)	<.001
Body mass index (BMI)	28.0 (25.8−31.2)	28.0 (25.2−32.0)	.11
Smoking			<.001
Never	39,737 (33%)	12,326 (47%)	
Exsmoker	66,211 (55%)	10,573 (40%)	
Current	15,371 (13%)	3258 (12%)	
Chronic Obstructive Pulmonary Disorder (COPD)	13,525 (11%)	3723 (14%)	<.001
Cerebrovascular disease			<.001
None	113,159 (93%)	24,151 (92%)	
Transient ischemic attack (TIA)	4342 (3.6%)	1082 (4.1%)	
Stroke with recovery	2368 (2.0%)	606 (2.3%)	
Stroke with deficit	1450 (1.2%)	318 (1.2%)	
Peripheral vascular disease (PVD)	14,661 (12%)	3292 (13%)	.025
Diabetes mellitus			<.001
None	85,551 (71%)	17,169 (66%)	
Diet controlled	5232 (4.3%)	1128 (4.3%)	
Tablet controlled	21,697 (18%)	4772 (18%)	
Insulin controlled	8839 (7.3%)	3088 (12%)	
Hypertension	91,150 (75%)	20,797 (80%)	<.001
Creatinine > 200 mmol/ml	1782 (1.5%)	319 (1.2%)	.002
Atrial fibrillation	4733 (3.9%)	772 (3.0%)	<.001
Recent myocardial infraction (1 month)	38,460 (32%)	9019 (34%)	<.001
Previous cardiac surgery	1688 (1.4%)	265 (1.0%)	<.001
Previous percutaneous coronary intervention			<.001
None	102,250 (84%)	22,239 (85%)	
<24 h, same admission	434 (0.4%)	87 (0.3%)	
>24 h, same admission	1613 (1.3%)	394 (1.5%)	
>24 h, previous admission	17,022 (14%)	3437 (13%)	
Left ventricular ejection fraction			<.001
Poor < 30%	5544 (4.6%)	905 (3.5%)	
Moderate 30%−50%	28,702 (24%)	5366 (21%)	
Good ≥ 50%	87,073 (72%)	19,886 (76%)	
New York Heart Association Score			<.001
1	35,885 (30%)	5989 (23%)	
2	59,911 (49%)	12,732 (49%)	
3	22,783 (19%)	6533 (25%)	
4	2740 (2.3%)	903 (3.5%)	
Canadian Cardiovascular Society Score			<.001
0	12,606 (10%)	2318 (8.9%)	
1	11,369 (9.4%)	1994 (7.6%)	
2	47,198 (39%)	9233 (35%)	
3	35,695 (29%)	8443 (32%)	
4	14,451 (12%)	4169 (16%)	
Euroscore II score	1.60 (1.17−2.35)	2.11 (1.46−3.15)	<.001
Urgency			<.001
Elective	71,235 (59%)	13,890 (53%)	
Urgent	50,084 (41%)	12,267 (47%)	

Abbreviations: CABG, coronary artery bypass graft; IQR, interquartile range.

^a^ Median (IQR); *n* (%).

^b^ Wilcoxon rank sum test; Pearson's *χ* ^2^ test.

**Table 2:** Operative characteristics of CABG

Characteristic	Male, *N* = 121,319^a^	Female, *N* = 26,157^a^	*p* Value^b^
Use of cardiopulmonary bypass	100,140 (86%)	21,383 (85%)	<.001
Aortic cross clamp time (mins)	52 (37, 62)	48 (34, 62)	<.001
Cardiopulmonary bypass time (mins)	82 (62, 103)	78 (58, 98)	<.001
Use of left internal mammary artery	110,028 (91%)	23,004 (88%)	<.001
Use of bilateral internal mammary arteries	6532 (5.4%)	820 (3.1%)	<.001
Use of right internal mammary artery	7226 (6.0%)	1025 (3.9%)	<.001
Use of radial artery	8269 (6.8%)	1621 (6.2%)	<.001
Vein grafts			<.001
Short saphenous	18,885 (16%)	4531 (17%)	
Long saphenous	101,909 (84%)	21,523 (82%)	
Other vein	525 (0.4%)	103 (0.4%)	
Total arterial grafts	15,040 (12%)	3716 (14%)	<.001
Number of grafts	3.00 (2.00−4.00)	3.00 (2.00−3.00)	<.001

Abbreviations: CABG, coronary artery bypass graft; IQR, interquartile range.

^a^ *n* (%); Median (IQR).

^b^ Pearson's *χ* ^2^ test; Wilcoxon rank sum test.

### 3.3 AVR

Twenty‐six thousand seven hundred forty‐two males (58.3%) and 19,168 females (41.7%) underwent an elective or urgent isolated AVR (see Table 3). Females tended to be older but were more likely to have an elective rather than urgent AVR compared to males (see supplementary table 1).

**Table 3:** Baseline characteristics of patients undergoing AVR, females compared to males

Characteristic	Male, *N* = 26,742^a^	Female, *N* = 19,168^a^	*p* Value^b^
Age (years)	70 (62−77)	74 (67−79)	<.001
Body mass index (BMI)	27.9 (25.2−31.1)	27.9 (24.6−32.5)	<.001
Smoking			<.001
Never	10,757 (40%)	11,195 (58%)	
Exsmoker	13,853 (52%)	6704 (35%)	
Current	2132 (8.0%)	1269 (6.6%)	
Chronic obstructive pulmonary disorder (COPD)	3512 (13%)	2981 (16%)	<.001
Cerebrovascular disease			<.001
None	24,149 (90%)	17,501 (91%)	
Transient ischemic attack (TIA)	1306 (4.9%)	959 (5.0%)	
Stroke with recovery	751 (2.8%)	457 (2.4%)	
Stroke with deficit	536 (2.0%)	251 (1.3%)	
Peripheral vascular disease (PVD)	2070 (7.7%)	1138 (5.9%)	<.001
Diabetes mellitus			.004
None	21,916 (82%)	15,756 (82%)	
Diet controlled	939 (3.5%)	727 (3.8%)	
Tablet controlled	3026 (11%)	2005 (10%)	
Insulin controlled	861 (3.2%)	680 (3.5%)	
Hypertension	16,918 (63%)	12,420 (65%)	<.001
Creatinine > 200 mmol/ml	676 (2.5%)	195 (1.0%)	<.001
Atrial fibrillation	3802 (14%)	2490 (13%)	<.001
Recent myocardial infraction (1 month)	549 (2.1%)	322 (1.7%)	.004
Previous cardiac surgery	2796 (10%)	1332 (6.9%)	<.001
Previous percutaneous coronary intervention			<.001
None	25,192 (94%)	18,495 (96%)	
<24 h, same admission	40 (0.1%)	33 (0.2%)	
>24 h, same admission	38 (0.1%)	26 (0.1%)	
>24 h, previous admission	1472 (5.5%)	614 (3.2%)	
Left ventricular ejection fraction			<.001
Poor < 30%	1632 (6.1%)	628 (3.3%)	
Moderate 30%−50%	5386 (20%)	2654 (14%)	
Good ≥ 50%	19,724 (74%)	15,886 (83%)	
New York Heart Association Score			<.001
1	3867 (14%)	1753 (9.1%)	
2	10,929 (41%)	7115 (37%)	
3	10,132 (38%)	8907 (46%)	
4	1814 (6.8%)	1393 (7.3%)	
Canadian Cardiovascular Society Score			.024
0	16,210 (61%)	11,453 (60%)	
1	3423 (13%)	2449 (13%)	
2	5053 (19%)	3651 (19%)	
3	1659 (6.2%)	1333 (7.0%)	
4	397 (1.5%)	282 (1.5%)	
Euroscore II score	1.67 (1.17−2.71)	1.99 (1.38−3.07)	<.001
Urgency			<.001
Elective	20,715 (77%)	15,649 (82%)	
Urgent	6027 (23%)	3519 (18%)	

Abbreviations: AVR, aortic valve replacement; IQR, interquartile range.

^a^ Median (IQR); *n* (%).

^b^ Wilcoxon rank sum test; Pearson's *χ* ^2^ test.

Females had shorter cross clamp times (65 vs. 70 min) and CPB times (87 vs 95 min) than males. Biological aortic valves were the most common implanted for both males and females however males were more likely to receive a mechanical implant (21% vs. 15%) or autologous graft (0.7% vs. 0.4%) than females.

### 3.4 MVR

Seven thousand nine hundred ninety‐one males (47.7%) and 8778 females (52.3%) underwent an elective or urgent isolated MVR (see Table 4). Females were more likely to have an elective rather than urgent MVR compared to males. Females tended to be older with higher rates of COPD and diabetes.

**Table 4:** Baseline characteristics of patients undergoing MVR, females compared to males

Characteristic	Male, *N* = 7991^a^	Female, *N* = 8778^a^	*p* Value^b^
Age (years)	68 (59−75)	69 (58−76)	<.001
Body mass index (BMI)	26.5 (23.7−29.1)	26.6 (23.1−30.3)	.037
Smoking			<.001
Never	3378 (42%)	4866 (55%)	
Exsmoker	3858 (48%)	2979 (34%)	
Current	755 (9.4%)	933 (11%)	
Chronic obstructive pulmonary disorder (COPD)	1071 (13%)	1620 (18%)	<.001
Cerebrovascular disease			<.001
None	7170 (90%)	7696 (88%)	
Transient ischemic attack (TIA)	345 (4.3%)	501 (5.7%)	
Stroke with recovery	257 (3.2%)	340 (3.9%)	
Stroke with deficit	219 (2.7%)	241 (2.7%)	
Peripheral vascular disease (PVD)	575 (7.2%)	473 (5.4%)	<.001
Diabetes mellitus			.009
None	6,956 (87%)	7,496 (85%)	
Diet controlled	212 (2.7%)	287 (3.3%)	
Tablet controlled	616 (7.7%)	726 (8.3%)	
Insulin controlled	207 (2.6%)	269 (3.1%)	
Hypertension	4342 (54%)	4586 (52%)	.007
Creatinine > 200 mmol/ml	318 (4.0%)	173 (2.0%)	<.001
Atrial fibrillation	2,865 (36%)	3,745 (43%)	<.001
Recent myocardial infraction (1 month)	383 (4.8%)	272 (3.1%)	<.001
Previous cardiac surgery	1555 (19%)	1412 (16%)	<.001
Previous percutaneous coronary intervention			.020
None	7543 (94%)	8373 (95%)	
<24 h, same admission	9 (0.1%)	12 (0.1%)	
>24 h, same admission	32 (0.4%)	24 (0.3%)	
>24 h, previous admission	407 (5.1%)	369 (4.2%)	
Left ventricular ejection fraction			<.001
Poor < 30%	537 (6.7%)	299 (3.4%)	
Moderate 30%−50%	2523 (32%)	2155 (25%)	
Good ≥ 50%	4931 (62%)	6324 (72%)	
New York Heart Association Score			<.001
1	923 (12%)	519 (5.9%)	
2	2662 (33%)	2408 (27%)	
3	3393 (42%)	4693 (53%)	
4	1013 (13%)	1158 (13%)	
Canadian Cardiovascular Society Score			.2
0	5404 (68%)	6044 (69%)	
1	956 (12%)	1066 (12%)	
2	1052 (13%)	1066 (12%)	
3	411 (5.1%)	431 (4.9%)	
4	168 (2.1%)	171 (1.9%)	
Euroscore II score	2.32 (1.45−4.13)	2.52 (1.60−4.29)	<.001
Urgency			<.001
Elective	5572 (70%)	6834 (78%)	
Urgent	2419 (30%)	1944 (22%)	

Abbreviations: IQR, IQR, interquartile range; MVR, mitral valve replacement/repair.

^a^ Median (IQR); *n* (%).

^b^ Wilcoxon rank sum test; Pearson's *χ* ^2^ test.

Females had shorter cross clamp times and CPB times than males. Males were more likely than females to have a MV repair rather than a MV replacement (see Supporting Information: Table 2).

### 3.5 Outcomes

**Figure 2:**
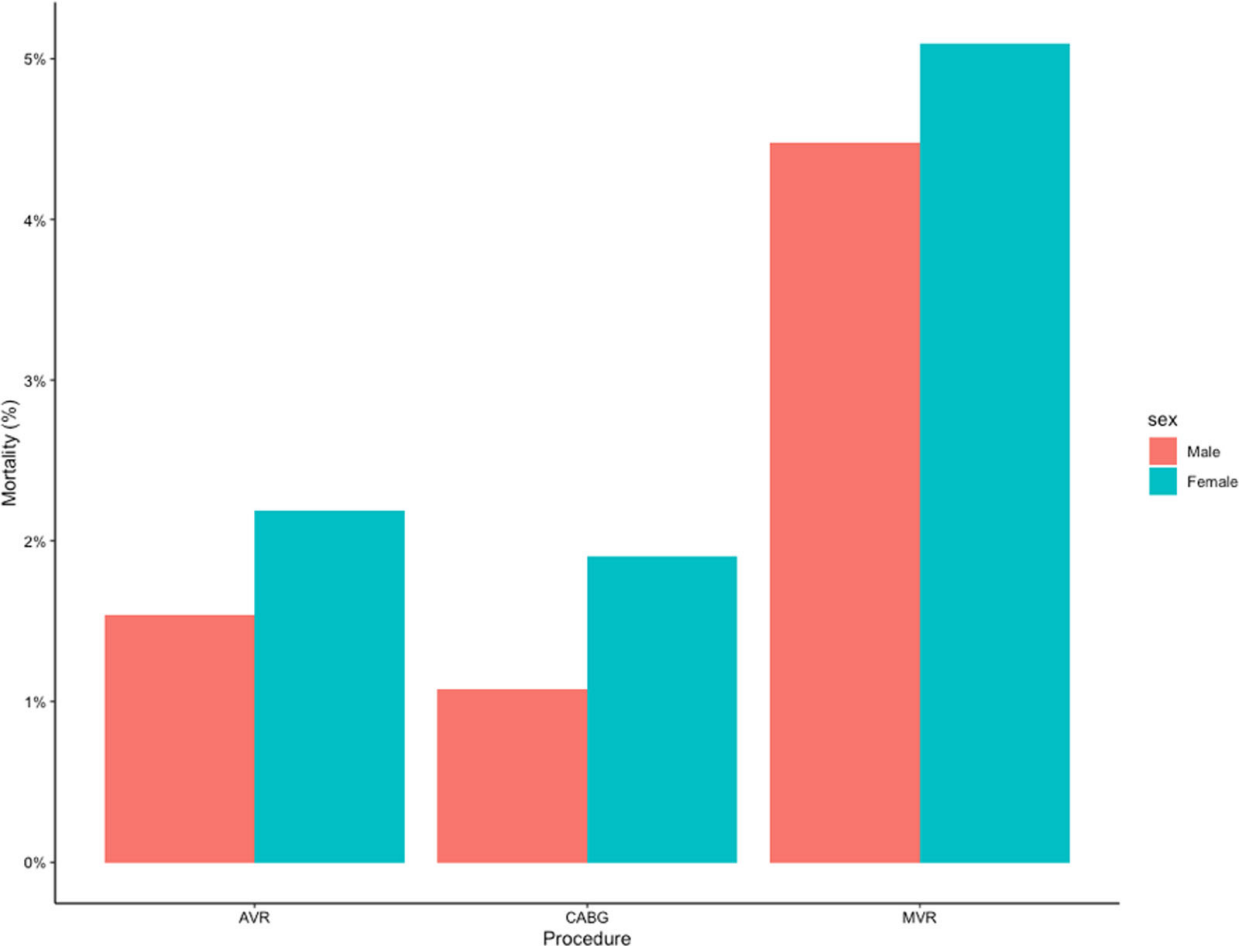
Percentage mortality of each sex by procedure AVR, CABG and MVR. AVR, aortic valve replacement; CABG, coronary artery bypass graft; MVR, mitral valve replacement/repair.

The rate of 30‐day mortality by procedure and within each sex stratum is presented in Figure 2.

**Figure 3:**
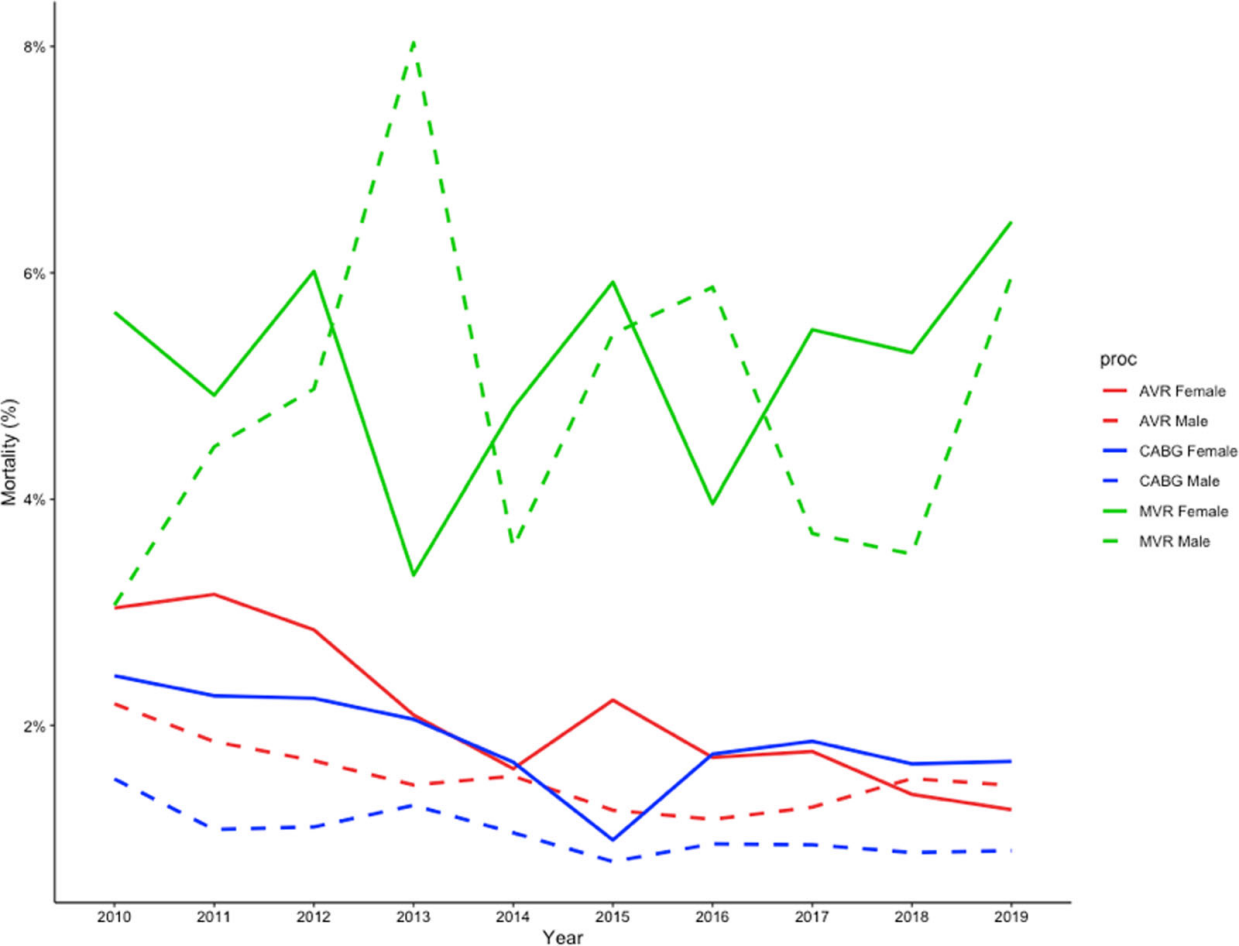
Percentage mortality of each sex by procedure AVR, CABG, and MVR over time. AVR, aortic valve replacement; CABG, coronary artery bypass graft; MVR, mitral valve replacement/repair.

Figure 3 shows 30‐day mortality by procedure and sex over time.

### 3.6 CABG

Females, compared to males, had greater odds of experiencing 30‐day mortality after CABG (OR 1.76, CI 1.47−2.09, *p* < .001) (see Table 5). Females also experienced increased need for postoperative dialysis (OR 1.31, CI 1.12–1.52, *p* < .001), deep sternal wound infections (OR 1.43, CI 1.11−1.83, *p* = .005) and length of stay (*β* 1.2, CI 1.0−1.4, *p* < .001) compared to males. However, female sex was protective against returning to theater for postoperative bleeding (OR 0.76, CI 0.65–0.87, *p* < .001). There was no sex‐related difference in postoperative cerebrovascular accident (CVA) following CABG (OR 1.01, CI 0.79−1.28, *p*> .9).

**Table 5:** Multivariable regression analysis of outcomes following CABG, females compared to males

Outcome	Males *N* = 121,3191	Females *N* = 26,1571	Adjusted OR	95% CI	*p* Value
Mortality	1302 (1.1%)	496 (1.9%)	1.76	1.47−2.09	<.001
Postoperative Cerebrovascular accident			1.01	0.79−1.28	>.9
Transient ischemic attack	397 (0.4%)	102 (0.4%)			
Stroke	558 (0.5%)	154 (0.7%)			
Return To theater for bleeding	3389 (2.8%)	619 (2.4%)	0.76	0.65, 0.87	<.001
Postoperative dialysis	1933 (1.7%)	574 (2.4%)	1.31	1.12−1.52	<.001
Deep sternal surgical site infection	695 (0.9%)	209 (1.3%)	1.43	1.11−1.83	.005
Hospital length of stay	8 (6−13)	9 (7−15)	1.2	1.0−1.4	<.001

Abbreviations: CABG, coronary artery by‐pass grafting; CI, confidence interval; OR, odd ratio.

### 3.7 AVR

Following AVR, females had a greater odds of 30‐day mortality compared to males (OR 1.59, CI 1.27−1.99, *p* < .001) (see Table 6). Female sex was protective against returning to theater for postoperative bleeding (OR 0.72, CI 0.61−0.84, *p* < .001) and deep sternal wound infections l SSI (OR 0.60, CI 0.33−1.03, *p* = .074). There were no significant gender‐related differences in outcomes of postoperative CVA (OR 1.03, CI 0.78−1.36, *p* = .8), postoperative dialysis (OR 0.94, CI 0.76−1.15, *p* = .5) and in hospital length of stay (*β* 0.2, CI −0.20 to 0.60, *p* = .3).

**Table 6:** Multivariable regression analysis of outcomes following AVR, females compared to males

Outcome	Males *N* = 26,7421	Females *N* = 19,1681	OR	95% CI	*p* Value
Mortality	525 (2.0%)	521 (2.7%)	1.59	1.27−1.99	<.001
Postoperative cerebrovascular accident			1.03	0.78−1.36	.8
Transient ischemic attack	180 (0.8%)	133 (0.8%)			
Stroke	224 (0.9%)	175 (1.0%)			
Return To theater for bleeding	1362 (5.1%)	681 (3.6%)	0.72	0.61−0.84	<.001
Postoperative dialysis	745 (3.1%)	487 (2.8%)	0.94	0.76−1.15	.5
Deep sternal surgical site infection	108 (0.6%)	61 (0.5%)	0.60	0.33−1.03	.074
Hospital length of stay	9 (7−14)	9 (7–15)	0.20	−0.20 to 0.60	.3

Abbreviations: AVR, aortic valve replacement; CI, confidence interval; OR, odd ratio.

### 3.8 MVR

Females had a greater odds of 30‐day mortality after MVR compared to males (OR 1.37, CI 1.09−1.71, *p* = .006) (see Table 7). However, females had less deep sternal wound infections (OR 0.56, CI 0.29−1.05, *p* = .076) and a reduced hospital LOS (*β* −1.6, CI −2.5 to −0.63, *p* < .001) compared to males. There were no significant differences between males and females for outcomes of postoperative CVA (OR 1.00, CI 0.71−1.43, *p*> .9), returning to theater for postoperative bleeding (OR 0.87, CI 0.70−1.08, *p* = .2) and postoperative dialysis (OR 0.88, CI 0.71−1.09, *p* = .3).

**Table 7:** Multivariable regression analysis of outcomes following MVR, females compared to males

Outcome	Males *N* = 79,911	Females *N* = 87,781	OR	95% CI	*p* Value
Mortality	456 (5.8%)	546 (6.3%)	1.37	1.09−1.71	.006
Postoperative cerebrovascular accident			1.00	0.71−1.43	>.9
Transient ischemic attack	75 (1.0%)	86 (1.1%)			
Stroke	123 (1.7%)	116 (1.5%)			
Return To theater for bleeding	550 (6.9%)	480 (5.5%)	0.87	0.70−1.08	.2
Postoperative dialysis	513 (7.0%)	489 (6.1%)	0.88	0.71−1.09	.3
Deep sternal surgical site infection	54 (1.2%)	45 (0.9%)	0.56	0.29−1.05	.076
Hospital length of stay	12 (8–22)	12 (9−20)	−1.6	−2.5 to −0.63	.001

Abbreviations: CI, confidence interval; MVR, mitral valve replacement/repair; OR, odd ratio.

## 4 DISCUSSION

This study is the first to review current practice of UK national data to compare sex‐related differences in outcomes following surgical coronary revascularisation and valvular cardiac procedures.

From our data set of 210,155 patients (25.7% female), we found female sex to be an important risk factor for 30 day mortality following CABG, AVR and MVR. Following CABG, female sex was also associated with increased postoperative need for dialysis, deep sternal wound infections, and length of hospital stay.

Of the 121,319 males (82.3%) and 26,157 females (17.7%) who underwent CABG, only 17.7% were female. It is well reported that females have more limited access to coronary revascularisation surgery. It is suggested that cardiovascular disease is recognized later in female and leads to delays in investigation and subsequent treatment.^3^ Whilst males are more likely to present with classical symptoms of cardiovascular disease, females often display different symptoms to those thought of as “typical”^14^ The present study supports the well reported claim that females undergoing coronary revascularisation surgery are often older and with more comorbidities than males.^3^ Furthermore, we found that women were also more likely to need urgent, as opposed to elective, revascularisation than men, which may be responsible for some of the poorer outcomes reported. Our findings somewhat agree with a nationwide study from Sweden that females had higher risk of mortality following CABG due to poorer preoperative risk profiles,^7^ however our study additionally found female sex was an independent risk factor after multivariable analysis. This difference may be due to the Sweden inclusion criteria of adults under 50 years of age whereas our study included all adult patients.

It is also suggested that sex‐related differences in operative strategy decisions and techniques may explain sex‐related differences in cardiac surgery outcomes.^14^ For example, a higher proportion of males compared with females received LIMA, RIMA, or BIMA grafting in both our cohort and other studies^15^ which is suggested to predispose females to incomplete myocardial revascularisation.^16^, ^17^ This finding is echoed in the United States STS data, from 2011 to 2019, showing that females are less likely to receive a LIMA to left anterior descending artery anastomosis.^18^

Nevertheless, the multivariable regression analysis used in our study adjusted for differences in baseline and operative differences, including revascularisation strategy and still a sex‐related difference remained in short‐term mortality. These findings suggest that female sex is an independent risk factor for short‐term mortality following CABG which supports the consensus of the current literature.^6^, ^19^ The idea of female sex being an independent risk factor for worse outcomes following CABG is speculated to be related to the more challenging anatomy of female patients, such as smaller coronary artery targets for grafting, narrower conduits and more diffuse patterns of coronary disease.^3^, ^20^ This is consistent with the findings of national level database review from Italy who ascribed the sex‐related difference in mortality to the differing operative strategies adopted for the more technically challenging coronary revascularisation of female patients.^9^

Our study also evaluated other post‐CABG outcomes. We did not find an increased risk of stroke following CABG as other national studies have reported.^8^, ^21^ This may be related to the fact that in our cohort of females a significant 15% underwent off pump revascularisation which has been reported to be particularly beneficial in women because of its effect to reduce the risk of stroke.^22^

Sternal wound complications were more common in females than males following CABG in our study. A risk prediction tool developed in the United Kingdom identifies female sex as one of six independent predictors of surgical site infection following cardiac surgery.^23^ The Barts Surgical Infection Ris score also includes raised BMI > 30, diabetes, left ventricular ejection fraction <45% and peripheral vascular disease; all of which were more common in our female patients. This finding may indicate a complex multifactorial impact of female sex on the risk of developing wound complications. This would suggest that efforts to prevent sternal wound infections should aim to target all of the these modifiable risk factors, especially in our female patients.

While the majority of patients who underwent CABG were male, single valve surgery was more evenly distributed between the sexes. In contrast to CABG, females were more likely to have a planned elective valve procedure. Despite this, female sex was associated with significantly higher short‐term mortality following both isolated AVR and MVR procedures.

Our finding of increased mortality following AVR in females is reflected from other nationally representative databases such as the USA^24^, ^25^ and a previous UK database analysis.^26^ Our findings of poorer preoperative health status in females was also shown in data from United States and similarly, even after adjusting for these differences, females still had higher mortality rates following AVR.^25^ However, other national studies did not report sex‐related differences in AVR mortality.^12^, ^27^

In our study, men were more likely to receive a mechanical aortic valve than women which may reflect the differences in age and comorbidities between the sexes at time of surgery and their influence on the management planning. It is known that women with severe aortic stenosis are diagnosed at a later stage of the disease process^28^ but even when adjusting for preoperative difference women are less likely to be referred for surgical AVR than men.^25^, ^29^

There is no clear explanation for why women have worse outcomes compared to men following AVR but several mechanisms have been implicated. For similar degrees of aortic stenosis, females tend to have higher transvalvular pressure gradients, thicker ventricle walls and smaller end‐systolic and end‐diastolic chamber sizes than males.^30^ Second, females on average receive smaller valves than males, the outcomes of patient‐prosthesis mismatch seem more severe in smaller size valves^31^ and therefore may effect women disproportionately. Furthermore, females are also more likely to require additional aortic annular enlargement than males leading to increased operative risk associated with the annular enlargement procedure.^5^

As with the other procedures, females in the United Kingdom experienced an increased odds of 30‐day mortality following MVR than males. A 2013 study of 3761 patients found a difference in mitral pathology between males and females undergoing mitral surgery; males were more likely to have mitral valve leaflet prolapse whereas females were more likely to have calcified mitral valve leaflets.^32^ This differences in pathology explains why females are more likely to need a mitral valve replacement whilst males are more likely to receive a mitral valve repair, a finding reiterated in our study. A study of MV procedures from United States, 2000−2009, also agreed men were more likely to receive a MV repair than women.^33^ This difference in surgical management strategy is thought to contribute to the poorer outcomes we see in females.^34^

Interestingly, for both CABG and AVR surgery, female sex seemed to be protective for postoperative bleeding resulting in returning to theater. Estrogen has a procoagulant effect which may confer benefit to limit postoperative bleeding.^35^ Despite females tending to have lower rates of returning to theater for bleeding, females have been shown to receive more postoperative red blood cell transfusions with males and this is associated with delayed recovery.^10^ Limitation of the present study include unknown transfusion rates and the inability to compare bleeding outcomes between pre and postmenopausal patients. These may serve as hypotheses for further research.

## 5 LIMITATIONS

The present study has several limitations. First, we are limited to short‐term outcomes without long‐term follow‐up. While, 30‐day outcomes following cardiac surgery are important for evaluation of safety and efficiency, this data only forms part of the story. It is often reported than sex ceases to be an independent determinant of outcomes following cardiac surgery in a long‐term follow‐up^36^ and it is important to know if this pattern is also reflected in the United Kingdom population and healthcare system. Second, this study used data from NACSA database and therefore we were limited to analyzing data collected for this purpose and as such data such as red blood cell transfusion was missing.

Lastly, by the nature of being an observational non‐randomized cohort, despite adjusting for known confounders, the effect of unknown confounders remains.

## 6 CONCLUSION

Despite advances in cardiac surgery, females in the United Kingdom have an increased risk of short‐term mortality after cardiac surgery compared to males. This highlights the need to focus on the understanding of the causes behind these disparities, followed by implementation of sex‐specific strategies to improve the outcomes of females undergoing cardiac surgery.

## AUTHOR CONTRIBUTIONS

**Lauren Kari Dixon**: Conceptualization, methodology, software, formal analysis, writing–original draft, writing−review and Editing. **Arnaldo Dimagli**: Conceptualization, methodology, software, formal analysis, writing−review and editing. **Ettorino Di Tommaso**: Conceptualization, formal analysis, writing−review and editing. **Shubhra Sinha**: Conceptualization, software, formal analysis, writing−review and editing. **Daniel Paul Fudulu**: Writing−review and editing. **Manraj Sandhu**: Writing−review and editing. **Umberto Benedetto**: Conceptualization, methodology, supervision. **Gianni Angelini**: Conceptualization, methodology, writing−review and editing, supervision.

## CONFLICT OF INTEREST

The authors declare no conflict of interest.

## ETHICS STATEMENT

All the analyzed data was anonymised; hence there was no need to obtain further ethics approval.

## Supporting information

Supplementary information.

Supplementary information.
