# Supplementary material for: Females have an increased risk of short‐term mortality after cardiac surgery compared to males: Insights from a national database
Source: J Card Surg. 2022 Sep 18;37(11):3507–19. doi: 10.1111/jocs.16928 (PMC9826035; doi:10.1111/jocs.16928)
Supplement: Supplementary file 1 — Supplementary information. [file JOCS-37-3507-s001.docx]

**Supplementary Material - Females have an increased risk of short-term mortality after cardiac surgery compared to males: Insights from a national database**

Contents:

Supplementary Table 1: Operative characteristics of AVR

Supplementary Table 2: Operative characteristics of MVR

Supplementary Table 1: Operative characteristics of AVR

| **Characteristic** | **Male**, N = 26,742*^1^* | **Female**, N = 19,168*^1^* | **p-value***^2^* |
| --- | --- | --- | --- |
| Aortic Cross Clamp Time (mins) | 70 (56, 91) | 65 (51, 85) | <0.001 |
| Cardiopulmonary Bypass Time (mins) | 95 (75, 121) | 87 (69, 112) | <0.001 |
| Aortic Stenosis Gradient | 68 (55, 83) | 69 (55, 85) | <0.001 |
| Aortic Effective Orifice Area | 0.80 (0.60, 0.90) | 0.70 (0.60, 0.80) | <0.001 |
| Aortic Valve Implant |  |  | <0.001 |
| Mechanical | 5,536 (21%) | 2,956 (15%) |  |
| Biological | 21,021 (79%) | 16,133 (84%) |  |
| Autologous graft | 185 (0.7%) | 79 (0.4%) |  |
| ^1^ Median (IQR); n (%)  ^2^ Wilcoxon rank sum test; Pearson's Chi-squared test | | | |

Supplementary Table 2: Operative characteristics of MVR

| **Characteristic** | **Male**, N = 7,991*^1^* | **Female**, N = 8,778*^1^* | **p-value***^2^* |
| --- | --- | --- | --- |
| Aortic Cross Clamp Time (mins) | 101 (74, 137) | 90 (66, 123) | <0.001 |
| Cardiopulmonary Bypass Time (mins) | 140 (106, 187) | 123 (94, 164) | <0.001 |
| Procedure |  |  | <0.001 |
| Replacement | 6,951 (87%) | 8,125 (93%) |  |
| Repair | 1,040 (13%) | 653 (7.4%) |  |
| ^1^ Median (IQR); n (%)  ^2^ Wilcoxon rank sum test; Pearson's Chi-squared test | | | |
